# Editorial: Nutrient metabolism and complications of type 2 diabetes mellitus

**DOI:** 10.3389/fnut.2026.1797839

**Published:** 2026-02-24

**Authors:** Yihan Fan, Wenting Geng, Ruihan Ma, Yan Gao, Jingliang Zhang, Renjun Lv, Bin Liu, Qingqing Yin

**Affiliations:** 1 Department of Geriatric Neurology, Shandong Provincial Hospital Affiliated to Shandong First Medical University, Jinan, Shandong, China; 2 Department of Neurology, The First Affiliated Hospital of Shandong First Medical University and Shandong Provincial Qianfoshan Hospital, Shandong Institute of Neuroimmunology, Jinan, Shandong, China; 3 Institute of Brain Science and Brain-Inspired Research, Shandong First Medical University and Shandong Academy of Medical Sciences, Jinan, Shandong, China; 4 Shandong Academy of Occupational Health and Occupational Medicine, Shandong First Medical University and Shandong Academy of Medical Sciences, Jinan, Shandong, China; 5 School of Clinical and Basic Medical Sciences, Shandong First Medical University and Shandong Academy of Medical Sciences, Jinan, Shandong, China

**Keywords:** abnormal bone metabolism, diabetic cognitive dysfunction, metabolic fatty liver disease, nutrition, nutritional and metabolic indicators

Type 2 diabetes mellitus (T2DM) is a highly prevalent metabolic disorder characterized by chronic hyperglycemia and pervasive dysregulation of nutrient metabolism. With disease progression, diabetes-related complications represent the major adverse outcomes, markedly compromising patients' quality of life and survival while placing a considerable burden on healthcare systems (1). Nutrient metabolism disorders are not only a core pathological feature of T2DM but also a key driver in the development of both microvascular and macrovascular complications (2, 3). Understanding the relationship between nutrient metabolism and diabetes complications, and identifying key nutrient metabolism factors influencing patient outcomes, is increasingly crucial for developing appropriate intervention strategies for patients with T2DM. Currently, there is extensive research on the relationship between nutrient metabolism and classic complications and comorbidities of diabetes (4). However, with longer disease durations and rising case numbers, the spectrum of diabetic complications has become far more intricate. Among these, non-classic complications such as cognitive dysfunction, abnormal bone metabolism, metabolic fatty liver disease, and sarcopenia are emerging as major factors affecting patient quality of life. To address this gap, this Research Topic presents studies that elucidate the link between nutrient metabolism and non-classic complications, highlighting the predictive value of emerging indicators, as well as potential strategies for personalized nutritional intervention and clinical management.

Medical nutrition therapy has a key role in T2DM management, with dietary interventions being central to improving glycaemic control and overall metabolic health. Recent evidence suggests that overall dietary patterns are more impactful than individual nutrients (5). El-Sehrawy et al. investigated the effects of the Baltic Sea Diet Score (BSDS) and the Healthy Nordic Diet Index (HNDI) on the risk of T2DM. This case-control study identified a significant inverse association between high adherence to the Nordic diet and the risk of developing T2DM. However, dietary interventions exhibit differential effects across diverse demographic groups. A significant finding of this Research Topic is the incorporation of sex-specific considerations into the analysis. Zhang, Meng et al., using National Health and Nutrition Examination Survey (NHANES) data (2001-2018), explored the impact of diet on cardiovascular (CV)/all-cause mortality in diabetic patients, and investigated whether this relationship changed by gender. High dietary scores, assessed by the Healthy Eating Index (HEI), the Alternative Healthy Eating Index (AHEI), and the alternative Mediterranean Diet (aMED) index, were strongly associated with lower CV/all-cause mortality in males but not in females. Among the component scores of the aMED, legume intake was unfavorable for males with diabetes but was remarkably associated with lower CV/all-cause mortality in females. Taken together, these findings highlight the central role of dietary management in T2DM care, while emphasizing that biological sex is an indispensable factor that cannot be overlooked.

It has become increasingly clear that nutrient metabolic imbalance acts as a molecular link connecting T2DM complications. We focus on the predictive and interventional value of nutritional and metabolic indicators in diabetic complications. Hung, Chang et al., through a retrospective cohort study, demonstrated that vitamin D deficiency (VDD) in newly diagnosed T2DM patients were independently associated with increased risk of diabetic retinopathy (DR) and other adverse outcomes, particularly in females. Two studies, by Chen, Zhang et al. and Chu et al., investigated the impact of the uric acid (UA) to high density lipoprotein cholesterol (HDL-C) ratio (UHR) on different diabetic complications. Chen, Zhang et al. conducted a cross-sectional study which revealed a significant dose-response relationship between the UHR and chronic kidney disease (CKD), suggesting its potential as an effective biomarker for CKD risk assessment. The identified UHR cut-off of 12.28 offered a practical threshold for early renal monitoring. Research in the field of non-classical complications has also explored the correlations between various nutritional indicators and disease. Chu et al. aimed to investigate the association between the UHR and mild cognitive impairment (MCI) in patients with T2DM. The results indicated that elevated UHR levels served as an independent risk factor for MCI in female T2DM patients, particularly regarding impairments in executive function and visuospatial abilities. However, no such significant association was observed in male patients. Regarding treatment, Cui et al. highlighted omega-3 polyunsaturated fatty acids as a potential adjunctive therapy for diabetes-associated cognitive dysfunction (DACD). Chen, Diao et al. performed a cross-sectional study that revealed an inverse association between the Healthy Eating Index (HEI-2020) and non-alcoholic fatty liver disease (NAFLD) risk in T2DM patients, an association that was partially mediated through the triglyceride-glucose (TyG) index and metabolic score (MS). Zhang, Jiang et al. demonstrated that obesity-related indicators and insulin resistance surrogates (IR surrogates) were significantly associated with osteoarthritis (OA) risk, with triglyceride-glucose index-waist-to-height ratio (TyG-WHtR) exhibiting the strongest predictive performance. In conclusion, these findings emphasize the significant potential of nutritional indices for the prediction, prevention, and management of T2DM complications.

In the era of digital medicine, technological innovations in big data and artificial intelligence serve as bridges connecting theory with clinical practice. Zhang, Lin et al. employed the XGBoost machine learning model to predict cardiovascular disease (CVD) risk in diabetic patients. By integrating dietary antioxidants with clinical variables, the model achieved a predictive accuracy of 87.4%. SHAP analysis clearly identified Daidzein and magnesium (Mg) as the most influential predictors.

In diabetes management, the value of nutritional intervention is evolving from simple glycemic control to risk stratification and precise intervention. Yu et al. conducted a systematic review highlighting the integration of systematic nutritional assessments and individualized treatments into multidisciplinary rehabilitation protocols. Integrating these with continuous glucose monitoring and machine learning analytics allowed clinicians to dynamically optimize macronutrient ratios, thereby significantly improving glycemic control, delaying complications, and enhancing functional recovery.

Overall, this Research Topic integrates evidence across dietary patterns, nutritional and metabolic indicators, and individualized management strategies, advancing our understanding of how nutrient metabolic dysregulation contributes to the development and progression of complications in T2DM. Emerging evidence underscores the critical influence of biological sex, age, and individual metabolic phenotypes on complication risk and intervention efficacy. By elucidating key nutrient metabolic factors underlying both classical and non-classical complications and translating these insights into precision, individualized management strategies, this Research Topic highlights a promising framework for reducing the overall burden of T2DM.
